# Dietary Magnesium May Be Protective for Aging of Bone and Skeletal Muscle in Middle and Younger Older Age Men and Women: Cross-Sectional Findings from the UK Biobank Cohort

**DOI:** 10.3390/nu9111189

**Published:** 2017-10-30

**Authors:** Ailsa A. Welch, Jane Skinner, Mary Hickson

**Affiliations:** 1 Department of Population Health and Primary Care, Norwich Medical School, Faculty of Medicine and Health Sciences, University of East Anglia, Norwich NR4 7TJ, UK; jane.skinner@uea.ac.uk; 2 Institute of Health and Community, Plymouth University, Peninsula Allied Health Centre, Derriford Road, Plymouth PL6 8BH, UK; mary.hickson@plymouth.ac.uk

**Keywords:** aging, skeletal muscle, grip strength, sarcopenia, physical function, bone mineral density, magnesium

## Abstract

Although fragility fractures, osteoporosis, sarcopenia, and frailty are becoming more prevalent in our aging society the treatment options are limited and preventative strategies are needed. Despite magnesium being integral to bone and muscle physiology, the relationship between dietary magnesium and skeletal muscle and bone health has not been investigated concurrently to date. We analysed cross-sectional associations between dietary magnesium and skeletal muscle mass (as fat free mass—FFM), grip strength, and bone density (BMD) in 156,575 men and women aged 39–72 years from the UK Biobank cohort. FFM was measured with bioelectrical impedance and was expressed as the percentage of body weight (FFM%) or as divided by body mass index (FFM_BMI_). Adjusted mean grip strength, FFM%, FFM_BMI_, and BMD were calculated according to quintiles of dietary magnesium, while correcting for covariates. Significant inter-quintile differences across intakes of magnesium existed in men and women, respectively, of 1.1% and 2.4% for grip strength, 3.0% and 3.6% for FFM%, 5.1% and 5.5% for FFM_BMI_, and 2.9% and 0.9% for BMD. These associations are as great or greater than annual measured losses of these musculoskeletal outcomes, indicating potential clinical significance. Our study suggests that dietary magnesium may play a role in musculoskeletal health and has relevance for population prevention strategies for sarcopenia, osteoporosis, and fractures.

## 1. Introduction

Fragility fractures, sarcopenia (the presence of low skeletal muscle mass and function), and frailty are becoming more prevalent in our aging society with their attendant disabilities and costs for health and social care. Moreover, maintaining mobility and wellbeing in our aging society is important. The costs of falls and fractures are £2.3 billion per year in the UK ($17 billion in the US), with one in two women and one in five men experiencing a fracture over the age of 50 years [1,2]. Estimates of the costs of sarcopenia are $US18.5B, and they are set to rise [3]. Osteoporosis (the presence of low bone density, BMD) is a well-recognised risk factor for fragility fractures [4,5,6,7], but more recently the age-related loss of skeletal muscle mass, function and sarcopenia, as well as frailty, have also been recognised as risk factors for osteoporosis, falls, and fractures [6,7,8]. Skeletal muscle provides protection through hormonal or endocrine interactions between muscle and bone, muscle force generated mechanical signals, and maintenance of postural balance. So, conserving skeletal muscle mass and function is important for prevention of fractures [9,10,11]. 

Both osteoporosis and sarcopenia are highly prevalent. Estimates for the prevalence of osteoporosis in the community are 22.1% and 5.5% in European women and men, aged 50 years and over [1]. In the USA 77.1% of women and 10.0% of men, over the age of 65 years have either osteoporosis or low bone density [12]. Sarcopenia has been identified in between 1% and 29% in community dwelling people over the age of 60 years, with estimates for those living in long term care of between 14% and 33% [13,14]. Recent predictions for Europe estimate that the prevalence of sarcopenia will almost double overall in the elderly population to 22.3% by 2045 [13,14]. The prevalence of frailty is 25% in those over the age in 80 [12]. It is clear that the prevalence of all these conditions (osteoporosis, sarcopenia and frailty, and number of fractures) will rise in line with the increasing age of populations in the UK and USA, as will the costs of health and social care for their treatment [3,13,15,16]. Prior to a diagnosis of these conditions, gradual losses in bone density and skeletal muscle mass and strength (sarcopenic risk factors) occur in a continuum starting from the age 30 years, with increasing rates of loss in those over the age of 60 years [17,18]. Importantly, these conditions, once present, are difficult to reverse and current treatment strategies are limited. Therefore, maintaining skeletal muscle and bone health during aging is important in our populations, and new preventative strategies in middle and younger older ages (middle age 40–60 years, younger older age 60 to 70 years) are needed.

Dietary composition can impact on the mechanisms leading to age-related loss of bone density, skeletal muscle mass or function. Calcium is well established as essential for bone health, as is protein for skeletal muscle, but other nutrients that are integral to bone and muscle physiology, such as magnesium (Mg), have not been investigated extensively and concurrently in relation to both skeletal muscle and bone health in both men and women of middle and younger older age [19,20,21,22,23]. 

The mechanism by which Mg may protect against osteoporosis and sarcopenic risk factors may be through the protection from cytokine induced stimulation of osteoclast activity or the protection of loss of skeletal muscle mass or strength. For osteoporosis, Mg can influence osteoblast activity as well as hydroxyapatite crystal formation, and regulation of calcium homeostasis through interactions between parathyroid hormone and vitamin D [24,25]. For skeletal muscle, Mg has direct physiological and metabolic roles, including maintenance of protein synthesis and turnover. Mg may also affect muscle performance though energy metabolism (production of ATP), transmembrane transport, and muscle contraction and relaxation [26,27]. Moreover, skeletal muscle and bone contain the majority of Mg in the body with 60% found in bone and 27% in muscle, indicating the importance of Mg to the musculoskeletal system [28].

Understanding the gender specific differences in associations between diet and skeletal muscle and bone health is important, as men attain a higher proportion of skeletal muscle mass, grip strength, and BMD at younger ages than women. Also, the effects of aging differ, with men losing a higher percentage of muscle mass and a lower percentage of BMD than women as they age. Few studies have investigated the sarcopenic risk factors with dietary magnesium in middle and older aged populations and even fewer investigated these associations according to gender. Grip strength has only been investigated in five previous studies with dietary or supplemental Mg intake or blood concentrations, and only one of these was in men [20,21,27,29,30]. Even fewer studies have investigated the associations between Mg and skeletal muscle mass, with none investigating associations in men only [20,21,29,30]. Of the greater number of studies investigating dietary magnesium and bone density, most were in older women (34), with only five investigating associations independently in men [25,31,32,33,34]. 

To our knowledge, no studies have previously investigated dietary Mg and measures of both bone and skeletal muscle health concurrently in the same cohort, independently in men and women. Therefore, we investigated the cross-sectional associations between dietary Mg intake and musculoskeletal health (skeletal muscle mass, hand grip strength and heel bone density) in middle and younger older aged men and women from the UK Biobank cohort, in a sample of 73,323 men and 82,098 women aged 39 to 72 years.

## 2. Materials and Methods 

### 2.1. Study Population

The United Kingdom (UK) Biobank cohort (application no. 11058) was used to study these associations. The UK Biobank is a prospective cohort study consisting of 502,655 people aged 37–73 years assessed between 2006 and 2010 in 22 assessment centres throughout the UK. The overall study UK Biobank Study received ethical approval from the North West Multi-centre Research Ethics Committee (reference number 06/MRE08/65). At recruitment, all of the participants gave informed consent to participate in UK Biobank and be followed-up, using a signature capture device. Further details of the rationale, design, and survey methods for UK Biobank have been published elsewhere [35].

### 2.2. Measurements of Body Composition Skeletal Muscle Mass, Grip Strength and Bone Density (Outcome Measures)

*Hand grip strength* was measured using a Jamar J00105 hydraulic hand dynamometer with three measurements made on the left hand and 3 made on the right hand side, which were then averaged [36,37]. The higher of these two measurements was used in the analyses [36,37].

*Height, weight, body composition and body mass index*. Standing height was measured using a Seca 202 height measure. Total body weight and fat-free mass (FFM), measured with bioelectrical impedance, were measured using the Tanita BC 418MA Body Fat Analyser [38]. Body mass index (BMI) was calculated as weight in kilograms divided by height squared in metres.

*Indices of fat free mass*. In order to control for increases in FFM with height and weight, the following indices were used [39]. Firstly, FFM as a percentage of body weight (FFM%), was calculated as total FFM (in kg divided by total body weight (in kg) multiplied by 100). Secondly, FFM divided by BMI (FFM_BMI_) was calculated since this takes into account the increase in body size, scaled for height, and it was calculated as total FFM divided by BMI [40]. Thirdly, total appendicular lean mass (ALM) was used because skeletal muscle mass in the limbs is more directly related to issues of mobility and the onset of sarcopenia was calculated as the sum of FFM in the arms and legs. ALM was scaled by BMI (ALM_BMI_) calculated as ALM divided by BMI [40].

*Bone Mineral Density*. The Sahara Clinical Bone Sonometer was used to estimate bone mineral density (BMD) based on ultrasound measurement of the calcaneus (heel) [41]. In the early stages of recruitment, only a single heel was used for the measurement, but in the later stages, measurements were made on both heels. The average of the two heel estimates was used in this study if both were available; otherwise, the single value was used (0.71% (*n* = 545) of the measurements that were used in our analyses were made the left had side only, and 0.76% (*n* = 581) of measurements were made on the right hand side only).

### 2.3. Measurement of Magnesium Intake

Dietary intake was assessed using the Oxford WebQ, a computerised 24-h recall questionnaire that was self-completed online on up to five occasions [42,43]. This questionnaire was designed to be completed on multiple occasions to reduce the potential measurement error that may occur with a single 24-h recall measurement. The Oxford WebQ questionnaire consists of 200 food items with associated choices of standard portion units or portion sizes [42,43]. This questionnaire has been validated against an interviewer-administered 24-h recall, with only small differences being found between the intakes of nutrients using both methods [43]. Intakes of nutrients from this questionnaire were calculated using composition data taken from McCance and Widdowson’s The Composition of Food and its supplements [43]. The Oxford WebQ was built in to the baseline assessments for the last 70,724 Biobank participants, and participants with a known e-mail address (66% of the cohort) were invited to complete it at a further four different time points over a 16-month interval at times designed to cover different week days and weekend days as well as seasonal variation [42]. Thus the Oxford WebQ was completed up to five times by participants. For those who completed it more than once (between two and five times), mean values of Mg intake were calculated. Of the individuals that were included in our analyses, 62% had completed more than one 24-hour recall. For the individuals used in this study, the maximum difference across months of the year for Mg was 2%, indicating minimal seasonal variation in the intakes of this nutrient. For this analysis, sex-specific quintiles of average Mg intake were used.

### 2.4. Measurement of Confounding Variables

Other variables included in the analysis were age group (39–44, 45–49, 50–54, 55–59, 60–64, 65–69, 70–72) and smoking status (never, previous/prefer not to say, or current). We calculated metabolic equivalents (METs) as the excess metabolic equivalent MET hours/week of physical activity during work and leisure time, as described in [44], and grouped participants into low (0 to <10 excess METs), moderate (10 to <50 excess METs) or high (≥50 excess METs) levels of physical activity. Energy, calcium, and vitamin D intakes from food were calculated as the average across the questionnaires that were completed. Protein intake was calculated as the percentage of average total energy intake from protein, and the models were adjusted for sex-specific quintiles of this. Binary variables for Mg, calcium, and vitamin D supplementation were derived from questions on supplement taking, including multivitamins and minerals. If a participant had answered that they had taken a relevant supplement on any of the food questionnaires, then this was coded as “yes”. To estimate potential misreporting of diet, the ratio of reported energy intake (EI) to estimated energy expenditure (EER) (EI:EER ratio), was calculated and adjusted for in the analyses [45]. The EER was calculated based on equations for men or women aged 19 years and older from the US Dietary Reference Intakes, and these equations were applied according to the BMI of participants; greater or less than 25 kg/m^2^ [45]. The equations used take into account age, height, and weight, as well as physical activity [45]. The number of dietary questionnaires completed was included as a covariate in analyses, as was self-reported use of cholesterol-lowering medication, and HRT (Hormone Replacement Therapy) use and menopausal status for women.

### 2.5. Study Participants

**Figure 1:**
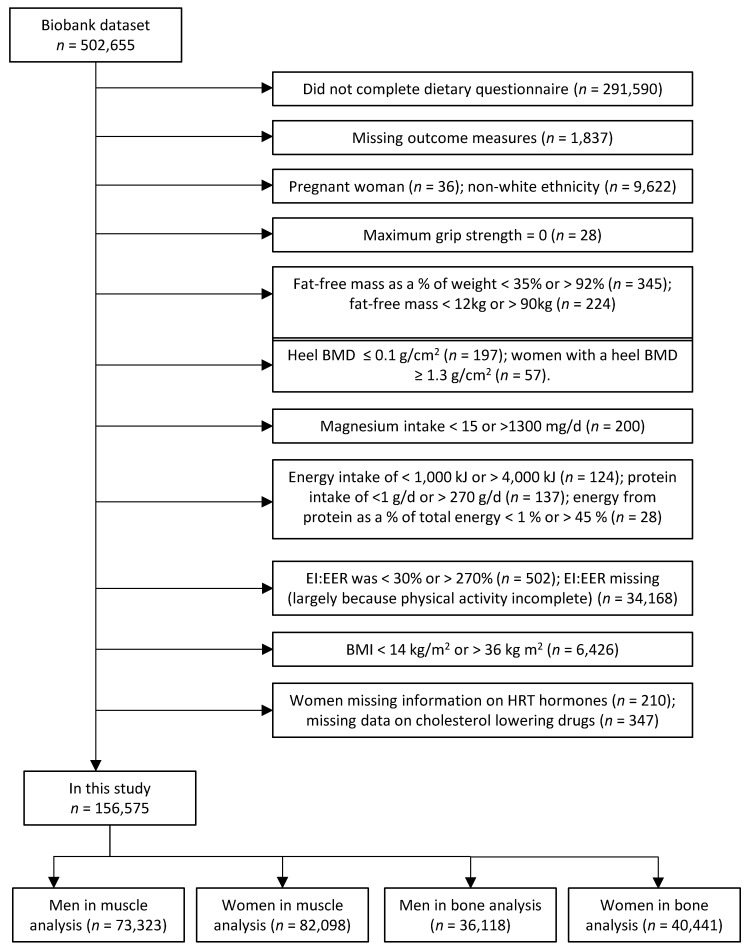
Flowchart of participants included in the study.

The Biobank dataset consisted of 502,655 people, however we excluded the following (see Figure 1): those without dietary or other relevant missing data, non-white ethnicity, pregnant women, those with a grip strength of zero, those with extremes of FFM, BMD, Mg, energy, protein, EI:EER, or BMI (bioelectrical impedance measures are considered unreliable at BMI extremes) [38]. These exclusions left a total of 156,575 people in the study (73,323 men and 82,098 women in the muscle analyses, 36,118 men and 40,441 women in the BMD analysis).

### 2.6. Statistical Analysis

We examined the association between dietary Mg, skeletal muscle mass and strength, and bone mineral density using multivariable regression techniques. First, for men and women separately, we calculated the mean and standard deviation of each outcome variable for each sex-specific quintile of dietary Mg (model 1). Then, we fitted Mg quintiles as the predictor in an adjusted model, again for men and women separately, with the covariates included. For this adjusted model, we calculated adjusted means with standard errors of each outcome for each sex-specific quintile of dietary Mg (model 2). For both models, we tested for a trend (*p* value given as *p trend* in the tables) in these unadjusted (model 1) and adjusted (model 2) values by fitting the median value of the outcome variable within each quintile as a continuous variable. Model 2 was also adjusted for sex-specific quintile of percentage energy from protein, smoking status, age group, physical activity levels, dietary energy intake, the ratio of EI:EER, the number of food questionnaires completed, whether the participant took Mg supplements, and, for women, whether the participant took HRT and whether she had experienced menopause. For the measures of skeletal muscle mass and strength, we also adjusted for whether the participant had taken cholesterol-lowering drugs. For BMD, we also adjusted for BMI, dietary calcium, dietary vitamin D, and calcium and vitamin D supplementation. For grip strength, we additionally adjusted for height. To determine whether there might be a different relationship between Mg and the outcomes of interest by age, we repeated the analyses stratified by age (<60 years and ≥60 years).

Loss of skeletal muscle can occur during the loss of body weight and also in certain conditions of chronic disease e.g., respiratory disease, diabetes, and chronic renal failure [46,47,48]. To test whether our analyses were affected by the loss of body weight that was linked to the presence of chronic illness, we performed a sensitivity analysis by dropping individuals from the study if they answered yes to both of two questions: (1) whether individuals had seen a weight change compared to a year ago and (2) whether they had a long-standing illness, disability or infirmity *n* = 7730. We then repeated the analyses on this smaller dataset (*n* = 148,845).

To understand the association between total fat free mass and total appendicular lean mass, the correlation was calculated in men and women.

In order to compare the relative scale of the associations between Mg intake and the different indices of skeletal muscle and bone, the differences in the values for these outcomes, between the top and bottom quintiles of Mg intake, were calculated as a percentage as follows; the difference between quintile 5 and quintile 1 of the values for the outcome indices were calculated and the percentage difference calculated as a percentage of the value of quintile 1. The statistical analyses were performed using STATA 14.0.

## 3. Results

The mean (SD) age of the men and women in this cohort was 56.7 (8.0) and 55.5 (7.8) years, respectively, with the majority of women being postmenopausal (69.4%) Table 1. As expected, women had a lower grip strength, proportion of total FFM, ALM, and BMD, expressed either as a percentage or in relation to BMI, than men, as shown in Table 1. For Mg, the mean and range of the intakes were also higher in men than women, and these intakes were higher than in the UK National Diet and Nutrition Survey (NDNS), a representative sample of the UK adults, of 268 mg/day in men and 212 mg/day in women. They were also similar to intakes in the EPIC-Norfolk study of 332 mg/day in men and 275 mg/day in women (aged 40–79 years), which were measured using 7-day diaries [25] Table 2. When compared with the dietary guidelines, intakes across the quintiles were all higher than the UK EAR of 250 mg/day in men and 200 mg/day in women [49]. However, intakes were lower than the more recent European Food Safety Authority recommendations for an Adequate Intake of 350 mg/day in men and 300 mg/day in women, in quintiles 1 and 2 [50]. Also, a small percentage of the population (2.2% of men and 1.1% of women) had intakes below the UK LRNI (Lower Reference Nutrient Intake) when compared with estimates from the NDNS of 12% in men and 11% in women aged 19–64 years [51]. We also note that there is substantial variation in Mg intake throughout Europe [50,52].

### 3.1. Grip Strength

Greater grip strength was associated with higher intakes of Mg with significant inter-quintile differences of 1.1% in men and 2.4% in women, after adjustment for covariates, representing differences of 0.5 kg and 0.6 kg in men and women, respectively (*p* for trend < 0.001), Table 2. After stratification for age, these inter-quintile differences were greater in older than in younger men; 1.7% (*p* trend = 0.001, men ≥ 60 years of age) versus 0.8% (*p* trend = 0.021, men < 60 years), Table 3. However, in women, the associations were stronger in younger than in older women; 2.5% versus 2.2% (*p* trend < 0.001), Table 3. 

To understand the clinical relevance of these associations, we compared our findings with dietary Mg with estimates of longitudinally measured loss of grip strength in men and women aged 75 years and over, which are 4% per year in men and 3% per year in women [17,53]. When comparing the magnitude of the interquintile differences in grip strength that is associated with magnesium intake with measured losses with age, our findings were about a quarter of the age-related losses in men and about three quarters of these losses in women (in men, the inter-quintile difference with Mg intake of 1.1% when divided by the previously measured loss per year of 4% equals one quarter (men 1.1%/4% = 0.25, for women 2.4%/3.0% = 0.8)).

### 3.2. Indices of Skeletal Muscle Mass

**Figure 2:**
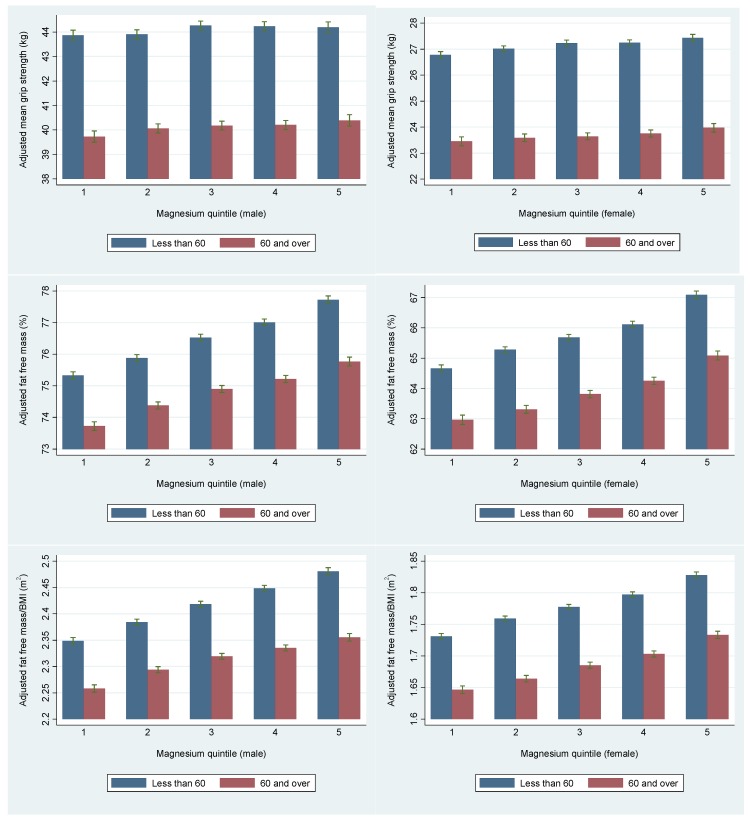

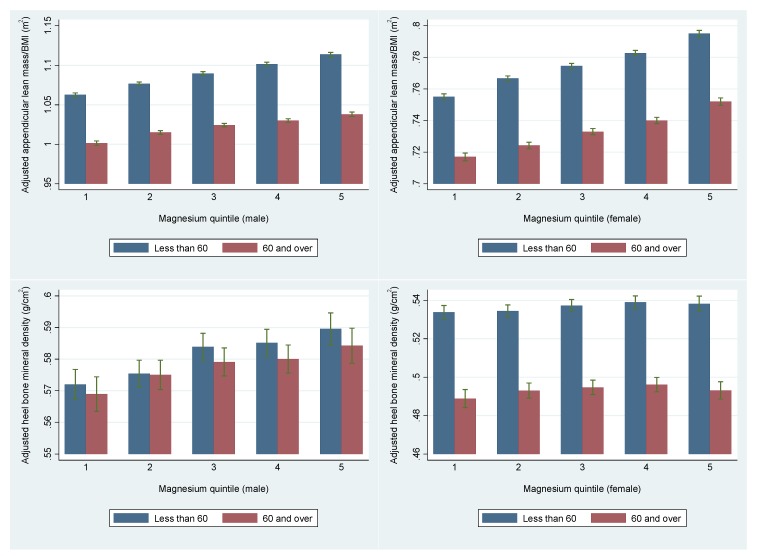
Associations between quintiles of magnesium intake and measurements of skeletal muscle mass and strength and bone density stratified by age above and below the age of 60 years.

All of the indices of skeletal muscle mass were positively associated with Mg intake in both of the unadjusted analyses, and in the analyses that were adjusted for covariates. The associations were of a similar scale for each index but were larger in women than in men. The inter-quintile differences in women were 3.6% for FFM%, 5.5% for FFM_BMI_, and 5.2% for ALM_BMI_, all *p* trend < 0.001, Table 2. In men, the corresponding inter quintile differences were 3.0% for FFM%, 5.1% for FFM_BMI_, and 4.4% for ALM_BMI_, all *p* trend < 0.001, Table 2. On stratification for age, in both men and women, the associations were of a smaller scale in older than in younger people, Table 3 and Figure 2. 

The correlation between total fat free mass and total appendicular lean mass was 0.97 (*p* < 0.001) in both men and women.

When we compared the interquintile differences with Mg with estimates of longitudinally measured loss of skeletal muscle mass of 1% per year in men and 0.7% per year for FFM%, these differences were 3 times that of yearly age related losses in men and 5.1 times in women (using our findings of inter-quintile differences of FFM% of 3.0% in men and 3.6% in women) [17,54]. This indicates the potential clinical significance of these associations.

### 3.3. Bone Mineral Density

Intakes of Mg were also associated with a greater BMD in men with significant inter-quintile differences of 2.9% in men (*p* trend < 0.001) and of 0.9% in women (*p* trend = 0.031), Table 2. These trends were similar in the age stratified analyses, with significant differences of 3.1% in younger (*p* trend < 0.001) versus 2.7% in older men (*p* trend = 0.001), Table 3, Figure 2. In women the differences in BMD were also significant but were similar (0.8% vs. 0.9%) in both age groups. When compared with the longitudinally measured annual loss of bone in men of 0.3% and 0.5% in women, our findings in men were 9.7 times larger than annual losses, and although the associations in women were smaller, they were still 1.8 times that of annual measured bone loss [33].

### 3.4. Sensitivity Analysis

In the results of the sensitivity analysis, to determine whether the weight loss associated with chronic disease conditions would impact on the main results, we found no differences in the associations compared with our main findings (data not shown). 

## 4. Discussion

This study extends scientific knowledge in this area as it is the first to investigate the associations between intakes of Mg concurrently with measurements of bone and skeletal muscle health in middle and older aged men and women. This is important as these factors are associated with an increased risk of falls, frailty, sarcopenia, and fractures. Higher intakes of dietary Mg were positively associated with a greater grip strength, indices of skeletal muscle mass, and BMD in both men and women aged 39–72 years continuously across the distribution of intakes. The inter-quintile differences associated with dietary Mg ranged from 1.1% and 2.4% for grip strength to 5.1% and 5.5% for FFM_BMI_, in men and women, respectively, but were smaller for BMD being 2.9% and 0.9%. When comparing these differences with previously measured longitudinal annual losses of skeletal muscle mass, grip strength and bone density, the associations found across the distribution of Mg intake ranged from one quarter in men to three quarters in women, for grip strength, and from 3 times in men to 5.1 times in women for FFM%. For BMD, these comparisons were 1.9 times in women and 9.7 times in men. Moreover, the associations were in the main of a similar scale in younger, as well as older, men and women, indicating that dietary Mg has relevance for protection for skeletal muscle and bone outcomes both in middle and younger older age groups. In men over the age of 60 years, the interquintile differences found in grip strength were around twice that of younger men. Whilst these findings are cross-sectional, they indicate that it is likely to be important for older men to consume sufficient dietary magnesium. Our findings were also significant after statistical adjustment for the important factors that contribute to skeletal muscle and bone loss: Age, smoking, and physical activity, and in women, HRT medication. For skeletal muscle our results remained significant after adjustment for dietary protein, which has been traditionally regarded as the most important nutrient for skeletal muscle health. For bone density, we also accounted for dietary and supplemental intakes of calcium. Thus, our results are independent of protein for muscle and of calcium for bone which have well established structural and physiological roles for musculoskeletal health. Mg also has important metabolic, physiological, and structural roles in the musculoskeletal system. As our findings imply that dietary Mg could have clinically relevant effects on skeletal muscle and bone health in both middle and older aged people, adequate dietary intakes of Mg are likely to be relevant for population prevention strategies.

The positive associations we found between grip strength and Mg in men and women in our study contrast with the two other cross-sectional studies and one intervention study [20,29,30,40]. These previous studies found no association with grip strength, despite one recent intervention study finding a significant effect of supplemental Mg on certain functional measures that were more pronounced in women with low intakes of Mg [20,21,29,30]. Another study found a positive association between serum Mg and hand grip strength [27], but serum Mg does not reflect dietary intake well, partly due to the tight homeostasis in blood, which is mediated by the reservoir of Mg within bone. Nevertheless, serum Mg is an integrated measure of dietary intake and a number of factors such as certain clinical conditions and medications [55].

The associations that we found between skeletal muscle mass (measured as FFM%) and intakes of Mg were a little lower than in a previous study of women, although in that study, FFM was measured using DXA, which is considered as a more precise method of measurement of body composition than bioelectrical impedance [21,56]. The only other study of which we are aware also found positive associations between skeletal muscle mass and dietary Mg analysed in men and women together [20]. We are unaware of data from other studies to compare our findings in only men, making this is the first study to investigate and find associations between dietary Mg and indices of skeletal muscle mass in men independently from women. For ALM, which is considered as an important measure of skeletal muscle that relates to risk of falls, our findings were similar to those that we found for the indices of total FFM. Moreover, total FFM and ALM were highly correlated in our study.

Heel BMD was also positively associated with dietary Mg in our study, with larger associations in men, which contrasts with the findings from a systematic review that found only small associations with dietary Mg and BMD of the femoral neck in the nine studies that were included. Overall, only five previous studies have examined intakes of Mg and bone density in men; all in smaller populations than this study, with only two finding significantly positive associations [25,31,32,33].

Although widely distributed in a range of foods, around 12% of middle and older aged people, in a UK national study, had intakes of Mg below the Lower Reference Nutrient Intake (LRNI) [49]. In our study, 2.2% of men and 1.1% of women consumed amounts of Mg below the LRNI, indicating individuals at risk of the symptoms of deficiency. However, even though intakes of Mg were higher than in the previous national study, the people in quintiles 1 and 2 (bottom 40% of the population) consumed intakes below the recommendations (EAR—estimated average requirement). Foods rich in Mg include nuts, whole grains and products, green leafy vegetables, berries, bananas, marine foods, and tap or bottled water that is high in Mg. Sufficient Mg in the diet can be achieved by following the UK and other government healthy eating guidelines [57] and our study further highlights the benefits of following these guidelines not only for cardiometabolic diseases, but also for musculoskeletal health.

We note that the women in this cohort reported a higher intake of energy as compared with predicted energy expenditure of 3.6%. This higher reporting of energy may be explained by the ‘frequency’ component of the Oxford Web Q, since frequency methods can produce higher estimates of intake than methods that are recorded over a period of time, such as seven day diaries [58]. Alternatively the women in this cohort may be consuming more energy than predicted from the equations that were used. Previous studies have indeed found that older women report greater amounts of energy intake than was predicted using equations [59].

Our study has a number of strengths, which include being the largest population to date to analyse dietary Mg intake concurrently with direct measures of skeletal muscle (as fat free mass), as well as bone health, independently in both men and women. This is particularly important due to the gender differences in attained skeletal muscle mass, grip strength, and BMD at younger age, and the differing effects of aging in men and women on these body systems. We also accounted for the established lifestyle and risk factors that are known to benefit measurements of skeletal muscle or bone density. We scaled our measurements of skeletal muscle mass for body weight or BMI to account for body size differences across the population. We also performed a sensitivity analysis to account for the potential effects of chronic conditions that are associated with weight loss on FFM, but this did not affect our findings.

One of limitations of this study is that it is a cross-sectional design and so we cannot infer causation. Also, since we excluded individuals of non-Caucasian background, our findings may not apply to those of different ethnic origin. Body composition was measured with BIA, which is considered as less precise than measurements made with DXA, although BIA is regarded as accurate in healthy individuals [56,60]. However, the method used for this study is single frequency BIA and so may underestimate the loss of skeletal muscle mass as compared with measurements made with multi-frequency BIA [61]. Heel BMD was measured by ultrasound attenuation rather than DXA, but previous studies have found that ultrasound methods are associated with osteoporotic risk factors and predict the incidence of fractures [62,63]. Although the self-reported measurements of physical activity we used are less precise than objective measures, they do distinguish across the range of activity levels for individuals [64].

## 5. Conclusions

Our research has found positive associations between greater intakes of dietary Mg and grip strength, indices of skeletal muscle mass, and BMD in men and women in middle and older age groups. These findings are of potential clinical significance when compared the annual losses of BMD and skeletal muscle with age. To our knowledge, this is the largest study to date to investigate dietary Mg with skeletal muscle, grip strength, and bone health in men and women independently. Our findings indicate that it is likely to be important to consume sufficient Mg as well as protein for the health of skeletal muscle, as well as calcium for bone. The results of our study suggest that dietary Mg may play a role in musculoskeletal health and have relevance for population prevention strategies for sarcopenia, frailty, falls, and fractures.

## Figures and Tables

**Table 1:** Characteristics and dietary intakes of the study subjects aged 39 to 72 years.

Characteristics	Men		Women	
	Muscle Measures Group (*n* = 73,323)	BMD Group (*n* = 36,118)	Muscle Measures Group (*n* = 82,098)	BMD Group (*n* = 40,441)
Age (years)	56.7 (8.0)	57.0 (8.1)	55.5 (7.8)	56.0 (8.0)
BMI (kg/m^2^)	27.0 (3.4)	27.1 (3.4)	25.7 (3.8)	25.8 (3.8)
Weight (kg)	84.4 (11.9)	84.7 (12.0)	68.7 (10.9)	68.9 (10.9)
Height (cm)	176.7 (6.6)	176.7 (6.6)	163.6 (6.1)	163.6 (6.1)
Hand-grip strength (kg)	42.3 (8.6)	41.3 (8.5)	25.9 (6.2)	25.1 (6.1)
Fat-free mass (FFM%)	75.7 (5.3)	75.5 (5.3)	65.1 (6.4)	64.7 (6.3)
FFM_BMI_	2.37 (0.26)	2.36 (0.26)	1.74 (0.21)	1.73 (0.21)
ALM_BMI_	1.06 (0.11)	1.06 (0.11)	0.76 (0.09)	0.76 (0.09)
Heel bone density (g/cm^2^)	0.580 (0.131)	0.579 (0.132)	0.519 (0.114)	0.519 (0.114)
Magnesium (mg/day)	371 (109)	371 (111)	335 (95)	334 (97)
Energy intake (kcal/day)	2301 (637)	2307 (651)	1967 (530)	1960 (538)
Protein (g/day)	87.3 (25.9)	87.4 (26.5)	78.3 (22.1)	78.0 (22.5)
Protein % energy	15.7 (3.4)	15.6 (3.5)	16.5 (3.7)	16.5 (3.7)
Misreporting (EI:EER, %)	89.8 (25.9)	89.7 (26.3)	103.6 (29.0)	103.0 (29.2)
No. of food recalls used	2.19 (1.18)	2.18 (1.27)	2.24 (1.19)	2.25 (1.29)
Physical activity				
low % (*n*)	24.1 (17,637)	23.0 (8292)	23.1 (18,943)	21.5 (8690)
moderate % (*n*)	54.5 (39,978)	53.6 (19,350)	56.8 (46,662)	56.3 (22,747)
high % (*n*)	21.4 (15,708)	23.5 (8476)	20.1 (16,493)	22.3 (9004)
Smoking status				
never % (*n*)	51.9 (38,024)	51.0 (18,404)	60.0 (49,258)	59.2 (23,931)
previous % (*n*)	39.3 (28,788)	39.6 (14,318)	33.4 (27,422)	33.8 (13,673)
current % (*n*)	8.9 (6511)	9.4 (3396)	6.6 (5418)	7.0 (2837)
Cholesterol-lowering drug % (*n*)	20.5 (14,994)	22.3 (8037)	8.8 (7183)	9.8 (3961)
Hormone-replacement therapy % (*n*)			7.7 (6340)	7.6 (3065)
Menopause % (*n*)			69.4 (56,956)	71.2 (28,782)

Values are mean (SD) unless stated as % (*n*). EI:EER = ratio of reported energy intake to estimated energy requirements, expressed as a percentage.

**Table 2:** Associations between quintiles of magnesium intake and measurements of skeletal mass and function and bone density.

**MEN**	**Model**	**Q1** **(*n* = 14,645)**	**Q2** **(*n* = 14,683)**	**Q3** **(*n* = 14,667)**	**Q4** **(*n* = 14,672)**	**Q5** **(*n* = 14,656)**	**Diff** **Q5-Q1**	**Q5-Q1/Q1 %**	***p*-trend**
Magnesium intake (mg/day)		238 ± 37	311 ± 15	359 ± 14	413 ± 18	532 ± 87	294	124	
Grip strength (kg)	1	41.9 ± 8.7	42.0 ± 8.4	42.3 ± 8.4	42.5 ± 8.5	42.9 ± 8.6	1.0	2.31	<0.001
	2	42.0 ± 0.08	42.2 ± 0.07	42.4 ± 0.07	42.4 ± 0.07	42.5 ± 0.08	0.5	1.09	<0.001
Fat free mass (%)	1	74.7 ± 5.2	75.3 ± 5.1	75.9 ± 5.2	76.2 ± 5.3	76.7 ± 5.5	2.0	2.69	<0.001
	2	74.6 ± 0.05	75.2 ± 0.04	75.8 ± 0.04	76.2 ± 0.04	76.9 ± 0.05	2.2	3.01	<0.001
Fat free mass_BMI_	1	2.31 ± 0.24	2.34 ± 0.25	2.37 ± 0.25	2.40 ± 0.26	2.43 ± 0.26	0.1	5.23	<0.001
	2	2.31 ± 0.002	2.34 ± 0.002	2.37 ± 0.002	2.40 ± 0.002	2.43 ± 0.002	0.1	5.10	<0.001
Appendicular lean mass_BMI_	1	1.04 ± 0.10	1.05 ± 0.10	1.06 ± 0.11	1.07 ± 0.11	1.08 ± 0.11	0.05	4.50	<0.001
	2	1.04 ± 0.001	1.05 ± 0.001	1.06 ± 0.001	1.07 ± 0.001	1.08 ± 0.001	0.05	4.37	<0.001
		**Q1** **(*n* = 7426)**	**Q2** **(*n* = 7112)**	**Q3** **(*n* = 7000)**	**Q4** **(*n* = 7194)**	**Q5** **(*n* = 7386)**			
Heel bone mineral density (g/cm^2^)	1	0.574 ± 0.133	0.577 ± 0.129	0.581 ± 0.132	0.582 ± 0.133	0.584 ± 0.131	0.01	1.61	<0.001
	2	0.570 ± 0.002	0.575 ± 0.002	0.582 ± 0.002	0.583 ± 0.002	0.587 ± 0.002	0.02	2.94	<0.001
**WOMEN**	**Model**	**Q1** **(*n* = 16,434)**	**Q2** **(*n* = 16,389)**	**Q3** **(*n* = 16,429)**	**Q4** **(*n* = 16,424)**	**Q5** **(*n* = 16,422)**	**Diff** **Q5-Q1**	**Q5-Q1/Q1 %**	***p*-trend**
Magnesium intake (mg/day)		217 ± 34	283 ± 13	326 ± 12	373 ± 16	476 ± 75	259	119	
Grip strength (kg)	1	25.6 ± 6.2	25.7 ± 6.1	25.9 ± 6.1	26.0 ± 6.2	26.1 ± 6.2	0.6	2.25	<0.001
	2	25.5 ± 0.05	25.7 ± 0.05	25.9 ± 0.04	26.0 ± 0.04	26.2 ± 0.05	0.6	2.40	<0.001
Fat free mass (%)	1	64.4 ± 6.3	64.7 ± 6.2	65.1 ± 6.3	65.3 ± 6.3	65.9 ± 6.6	1.5	2.39	<0.001
	2	64.0 ± 0.05	64.6 ± 0.04	65.0 ± 0.04	65.4 ± 0.04	66.3 ± 0.05	2.3	3.62	<0.001
Fat free mass_BMI_	1	1.71 ± 0.21	1.73 ± 0.21	1.74 ± 0.21	1.76 ± 0.21	1.78 ± 0.22	0.1	4.46	<0.001
	2	1.70 ± 0.002	1.72 ± 0.002	1.74 ± 0.002	1.76 ± 0.002	1.79 ± 0.002	0.1	5.52	<0.001
Appendicular Lean Mass_BMI_	1	0.74 ± 0.09	0.75 ± 0.09	0.76 ± 0.09	0.77 ± 0.09	0.78 ± 0.09	0.03	4.20	<0.001
	2	0.74 ± 0.001	0.75 ± 0.001	0.76 ± 0.001	0.77 ± 0.001	0.78 ± 0.001	0.04	5.18	<0.001
		**Q1** **(*n* = 8302)**	**Q2** **(*n* = 8092)**	**Q3** **(*n* = 8046)**	**Q4** **(*n* = 7941)**	**Q5** **(*n* = 8060)**			
Heel bone mineral density (g/cm^2^)	1	0.519 ± 0.113	0.519 ± 0.114	0.520 ± 0.112	0.520 ± 0.115	0.517 ± 0.115	0.00	−0.56	0.189
	2	0.516 ± 0.001	0.518 ± 0.001	0.520 ± 0.001	0.522 ± 0.001	0.520 ± 0.002	0.00	0.85	0.031

Values for Model 1 are mean ± SD, for Model 2 are adjusted mean ± SE. Model 1 is unadjusted. Model 2 is adjusted (for all outcomes) for quintile of percentage energy from protein, smoking status, age group, physical activity levels, dietary energy intake, the ratio EI:EER, the number of food questionnaires completed, whether the participant took magnesium supplements and, for women, whether the participant took Hormone Replacement Therapy (HRT) and whether she had had menopause. For the measures of muscle mass and strength, model 2 is adjusted for whether the participant had taken cholesterol-lowering drugs. For the measure of heel bone mineral density, model 2 is adjusted for BMI, dietary calcium, dietary vitamin D, and calcium and vitamin D supplementation. For the grip strength outcome, height is additionally adjusted for.

**Table 3:** Associations between quintiles of magnesium intake and measurements of skeletal mass and function and bone density stratified by age.

**MEN**	**Subjects** **Age < 60** **Age ≥ 60**	**Q1** **(*n* = 8597)** **(*n* = 6048)**	**Q2** **(*n* = 8063)** **(*n* = 6620)**	**Q3** **(*n* = 7841)** **(*n* = 6826)**	**Q4** **(*n* = 7973)** **(*n* = 6699)**	**Q5** **(*n* = 8292)** **(*n* = 6364)**	**Diff** **Q5-Q1**	**Q5-Q1/Q1 %**	***p*-trend**
Magnesium intake (mg/day)	Age < 60	237 ± 37	310 ± 15	359 ± 14	413 ± 18	535 ± 90	298	126	
	Age ≥ 60	240 ± 36	311 ± 15	359 ± 14	413 ± 18	529 ± 84	289	120	
Grip strength (kg)	Age < 60	43.9 ± 0.10	43.9 ± 0.09	44.3 ± 0.09	44.2 ± 0.09	44.2 ± 0.11	0.3	0.75	0.021
	Age ≥ 60	39.7 ± 0.12	40.1 ± 0.10	40.2 ± 0.09	40.2 ± 0.09	40.4 ± 0.12	0.7	1.67	0.001
Fat free mass (%)	Age < 60	75.3 ± 0.06	75.9 ± 0.05	76.5 ± 0.05	77.0 ± 0.05	77.7 ± 0.06	2.4	3.19	<0.001
	Age ≥ 60	73.7 ± 0.07	74.4 ± 0.06	74.9 ± 0.06	75.2 ± 0.06	75.8 ± 0.07	2.0	2.77	<0.001
Fat free mass_BMI_	Age < 60	2.35 ± 0.003	2.38 ± 0.003	2.42 ± 0.003	2.45 ± 0.003	2.48 ± 0.003	0.1	5.64	<0.001
	Age ≥ 60	2.26 ± 0.004	2.29 ± 0.003	2.32 ± 0.003	2.34 ± 0.003	2.36 ± 0.004	0.1	4.31	<0.001
Appendicular Lean Mass_BMI_	Age < 60	1.06 ± 0.001	1.08 ± 0.001	1.09 ± 0.001	1.10 ± 0.001	1.11 ± 0.001	0.05	4.82	<0.001
	Age ≥ 60	1.00 ± 0.001	1.01 ± 0.001	1.02 ± 0.001	1.03 ± 0.001	1.04 ± 0.001	0.04	3.66	<0.001
	**Subjects** **Age < 60** **Age ≥ 60**	**Q1** **(*n* = 4146)** **(*n* = 3280)**	**Q2** **(*n* = 3732)** **(*n* = 3380)**	**Q3** **(*n* = 3569)** **(*n* = 3431)**	**Q4** **(*n* = 3672)** **(*n* = 3522)**	**Q5** **(*n* = 3938)** **(*n* = 3448)**			
Heel bone mineral density (g/cm^2^)	Age < 60	0.572 ± 0.002	0.575 ± 0.002	0.584 ± 0.002	0.585 ± 0.002	0.590 ± 0.003	0.02	3.07	<0.001
	Age ≥ 60	0.569 ± 0.003	0.575 ± 0.002	0.579 ± 0.002	0.580 ± 0.002	0.584 ± 0.003	0.02	2.69	0.001
**WOMEN**	**Subjects** **Age < 60** **Age ≥ 60**	**Q1** **(*n* = 11,128)** **(*n* = 5306)**	**Q2** **(*n* = 10,440)** **(*n* = 5949)**	**Q3** **(*n* = 10,305)** **(*n* = 6124)**	**Q4** **(*n* = 10,051)** **(*n* = 6373)**	**Q5** **(*n* = 9799)** **(*n* = 6623)**	**Diff** **Q5-Q1**	**Q5-Q1/Q1 %**	***p*-trend**
Magnesium intake (mg/day)	Age < 60	216 ± 35	283 ± 13	326 ± 12	373 ± 16	476 ± 76	260	120	
	Age ≥ 60	219 ± 32	283 ± 13	326 ± 12	373 ± 16	475 ± 75	256	117	
Grip strength (kg)	Age < 60	26.8 ± 0.06	27.0 ± 0.06	27.2 ± 0.06	27.2 ± 0.06	27.4 ± 0.07	0.7	2.46	<0.001
	Age ≥ 60	23.5 ± 0.09	23.6 ± 0.07	23.6 ± 0.07	23.8 ± 0.07	24.0 ± 0.08	0.5	2.21	<0.001
Fat free mass (%)	Age < 60	64.7 ± 0.06	65.3 ± 0.05	65.7 ± 0.05	66.1 ± 0.05	67.1 ± 0.06	2.4	3.76	<0.001
	Age ≥ 60	63.0 ± 0.08	63.3 ± 0.07	63.8 ± 0.06	64.3 ± 0.06	65.1 ± 0.07	2.1	3.37	<0.001
Fat free mass_BMI_	Age < 60	1.73 ± 0.002	1.76 ± 0.002	1.78 ± 0.002	1.80 ± 0.002	1.83 ± 0.003	0.1	5.62	<0.001
	Age ≥ 60	1.65 ± 0.003	1.66 ± 0.003	1.69 ± 0.002	1.70 ± 0.002	1.73 ± 0.003	0.1	5.28	<0.001
Appendicular Lean Mass_BMI_	Age < 60	0.76 ± 0.001	0.77 ± 0.001	0.77 ± 0.001	0.78 ± 0.001	0.80 ± 0.001	0.04	5.30	<0.001
	Age ≥ 60	0.72 ± 0.001	0.72 ± 0.001	0.73 ± 0.001	0.74 ± 0.001	0.75 ± 0.001	0.04	4.89	<0.001
	**Subjects** **Age < 60** **Age ≥ 60**	**Q1** **(*n* = 5325)** **(*n* = 2977)**	**Q2** **(*n* = 4874)** **(*n* = 3218)**	**Q3** **(*n* = 4754)** **(*n* = 3292)**	**Q4** **(*n* = 4597)** **(*n* = 3344)**	**Q5** **(*n* = 4550)** **(*n* = 3510)**			
Heel bone mineral density (g/cm^2^)	Age < 60	0.534 ± 0.002	0.535 ± 0.002	0.537 ± 0.002	0.539 ± 0.002	0.538 ± 0.002	0.00	0.83	0.070
	Age ≥ 60	0.489 ± 0.002	0.493 ± 0.002	0.495 ± 0.002	0.496 ± 0.002	0.493 ± 0.002	0.00	0.87	0.256

Values for Model 1 are adjusted mean ± SE. Models are adjusted (for all outcomes) for quintile of percentage energy from protein, smoking status, age group, physical activity levels, dietary energy intake, the ratio EI:EER, the number of food questionnaires completed, whether the participant took magnesium supplements and, for women, whether the participant took HRT and whether she had had menopause. For the measures of muscle mass and strength, the models are adjusted for whether the participant had taken cholesterol-lowering drugs. For the measure of heel bone mineral density, the model is adjusted for BMI, dietary calcium, dietary vitamin D, and calcium and vitamin D supplementation. For the grip strength outcome, height is additionally adjusted for. A test for trend was carried out by fitting the median value of the outcome variable within each quintile as a continuous variable.
